# Developmental exposure to the SSRI citalopram causes long-lasting behavioural effects in the three-spined stickleback (*Gasterosteus aculeatus*)

**DOI:** 10.1007/s10646-017-1866-4

**Published:** 2017-10-23

**Authors:** M. Kellner, T. Porseryd, I. Porsch-Hällström, B. Borg, C. Roufidou, K. H. Olsén

**Affiliations:** 1 School of Natural Sciences, Technology and Environmental Studies, Södertörn University, Alfred Nobels allé 7, SE-141 89 Huddinge, Sweden; 2 Department of Zoology, Stockholm University, Svante Arrhenius väg 18 B, SE-106 91 Stockholm, Sweden

**Keywords:** SSRI, Fish, Scototaxis, Locomotor, Aggression, Feeding

## Abstract

Selective Serotonin re-uptake inhibitors (SSRIs) are a class of psychotropic drugs used to treat depression in both adolescents and pregnant or breast-feeding mothers as well as in the general population. Recent research on rodents points to long-lasting behavioural effects of pre- and perinatal exposure to SSRIs which last into adulthood. In fish however, studies on effects of developmental exposure to SSRIs appears to be non-existent. In order to study effects of developmental SSRI exposure in fish, three-spine sticklebacks were exposed to 1.5 µg/l of the SSRI citalopram in the ambient water for 30 days, starting two days post-fertilisation. After approximately 100 days of remediation in clean water the fish were put through an extensive battery of behavioural tests. Feeding behaviour was tested as the number of bites against a piece of food and found to be increased in the exposed fish. Aggression levels were measured as the number of bites against a mirror image during 10 min and was also found to be significantly increased in the exposed fish. Novel tank behaviour and locomotor activity was tested in an aquarium that had a horizontal line drawn half-way between the bottom and the surface. Neither the latency to the first transition to the upper half, nor the number of transitions or the total time spent in the upper half was affected by treatment. Locomotor activity was significantly reduced in the exposed fish. The light/dark preference was tested in an aquarium where the bottom and walls were black on one side and white on the other. The number of transitions to the white side was significantly reduced in the exposed fish but there was no effect on the latency to the first transition or the total time spent in the white half. The results in the current study indicate that developmental SSRI exposure causes long-lasting behavioural effects in fish and contribute to the existing knowledge about SSRIs as environmental pollutants.

## Introduction

Selective serotonin re-uptake inhibitors (SSRIs) are a group of anti-depressants which act on the evolutionarily ancient and highly conserved serotonergic system. They exert their effect by inhibiting the re-uptake of serotonin (5-HT) into the pre-synaptic nerve terminal, thus causing an elevated level of serotonin in the synaptic cleft. SSRIs are typically lipophilic (Kwon and Armbrust 2008), have a relatively slow elimination rate (Kwon and Armbrust 2005; Bergersen, Hanssen, and Vasskog 2012) and are only broken down to a limited extent in sewage treatment plants (Vasskog et al. 2006; Yuan et al. 2013). For those reasons and because they are specifically designed to have behavioural effects, they have attracted interest as environmental pollutants. In this study citalopram was chosen as model SSRI. Citalopram was chosen partly because in Sweden and large parts of the world, it is the most frequently used SSRI but also because it is generally regarded as the most selective SSRI (Sánchez and Hyttel 1999), thus as far as possible excluding effects on other neuroendocrine systems than the serotonergic system. Citalopram has been found in sewage treatment plant effluents in concentrations ranging from 9.2 ng/l (Vasskog et al. 2006) to 720 ng/l (Wahlberg et al. 2008). In surface waters it has been found in a range between 4 ng/l (Giebułtowicz and Nałęcz-Jawecki 2014) and 76 µg/l although the latter is an extreme case from a site 150 m downstream a sewage treatment plant in India which receives large amounts of wastewater from drug manufacturers (Fick et al. 2009). More typical loads are in the 10–150 ng/l range while the predicted concentration needed in surrounding water to reach human therapeutic levels in fish is 141 ng/l for citalopram (Fick et al. 2010). Since there are several SSRIs in active use however, the total SSRI concentration may be much higher than the concentration of a single drug will show (Schlüsener et al. 2015; Schultz et al. 2010; Vasskog et al. 2008). In addition, some of the SSRI metabolites retain some SSRI effect (Hiemke and Härtter 2000; Pawlowski et al. 1985), adding to the total SSRI concentration.

SSRIs act on the highly complex serotonergic system which is involved in the regulation of mood and a range of different behaviours (Sánchez 2004). In addition to its properties as a modulator of behaviour, serotonin in mammals modulates neuronal outgrowth (Fricker et al. 2005) and genetically modified hyposerotonergic mice often suffer from various mood disorders as well as impaired growth and thermoregulation (Trowbridge, Narboux-Nême, and Gaspar 2011). At the other end of the spectrum, an increased serotonergic tone caused by reduced expression of the serotonin re-uptake transporter (5-HTT) predisposes individuals for anxiety-like behavioural traits (Holmes et al. 2003; Lesch et al. 1996). In adult mice, such behaviour can be mimicked by exposing them to the serotonin re-uptake inhibitor (SSRI) fluoxetine during early post-natal development (Ansorge 2004), indicating that exposure in early life disrupts serotonergic system development.

In mammals, often studied effects of SSRI on individuals exposed as adults, are reduced levels of anxiety (Mombereau et al. 2010; Sanchez et al. 2003), altered feeding behaviour (Rozenblit-Susan et al. 2016) and reduced levels of aggression (Caldwell and Miczek 2007). A wide range of literature on SSRI effects in fish paints a similar picture, with exposed animals exhibiting a behavioural phenotype characterised by reduced anxiety and stress response. It is beyond the scope of this paper to cover all of this literature but excellent reviews are found in Kreke and Dietrich (2008), Brooks (2014). Examples of frequently reported effects are anxiolysis (Kellner et al. 2016; Sackerman et al. 2010; Egan et al. 2009; Maximino et al. 2010), diminished response to alarm substances (Barbosa et al. 2012; Barry 2012), suppressed feeding behaviour (Kellner et al. 2015; Weinberger and Klaper 2014; Mennigen et al. 2009) and reduced levels of aggression (Dzieweczynski and Hebert 2012; Kohlert et al. 2012). Thus, using the novel tank test, Kellner et al. (2016) found that a three week exposure to 1.5 and 15 µg/l citalopram had anxiolytic effects on the three-spine stickleback, Sackerman et al. (2010) found that a 3 min exposure to 100 mg/l of citalopram had anxiolytic effects on zebrafish and Egan et al. (2009) found that 100 µg/l of fluoxetine had anxiolytic effects on zebrafish. Maximino et al. (2010) found anxiolytic effects in zebrafish after injections of 10 mg/kg b.w. fluoxetine using the scototaxis test. Barbosa et al. (2012) found diminished reactions to alarm substances measured as freezing, propensity of the fish to stay close to the bottom and increased school cohesion in piauçu fish after injections with 10 µg/g b.w. fluoxetine and Barry (2012) reported effects of several different concentrations of fluoxetine on swimming speed and school cohesion after alarm substance exposure in Arabian killifish. Effects on feeding behaviour were found by Kellner et al. (2015) in three-spine sticklebacks, measured as the number of feeding strikes, after 3 weeks of exposure to 1.5 µg/l of citalopram. Weinberger and Klaper found effects on feeding measured as changes in feeding rate after four weeks of exposure to 10 and 100 µg/l fluoxetine and Mennigen et al. (2009) found effects on food intake and weight gain in goldfish after a 13 day period of injections every third day with 5 µg/g b.w. of fluoxetine. Dziewezynski and Hebert (2012) has reported reduced levels of aggression in siamese fighting fish, measured as the number of attacks against a dummy conspecific after 5 h of exposure to 0.54 µg/l fluoxetine. Decreased aggression was also reported as measured by the number of attacks against a mirror image in siamese fighting fish after 18 days of exposure to 350 or 705 µg/l of fluoxetine by Kohlert et al. (2012).

While studies investigating survival and behaviour of young fish under SSRI exposure have been performed (Pelli and Connaughton 2015), studies on effects of developmental exposure to SSRI in adult fish are, to the best knowledge of the authors, completely absent. In rodents, individuals exposed to SSRI during development often exhibit an altered adult behavioural phenotype which includes anxious response to novelty (Ansorge 2004; Iñiguez et al. 2010; Rodriguez-Porcel et al. 2011; Simpson et al. 2011) and altered social behaviour such as reduced juvenile play behaviour (Simpson et al. 2011; Rodriguez-Porcel et al. 2011), reduced conspecific interaction (Rodriguez-Porcel et al. 2011) and increased aggressive behaviour (Kiryanova et al. 2016). Developmental SSRI exposure can also suppress feeding behaviour in novelty-suppressed feeding tests (Ansorge 2004; Iñiguez et al. 2010), and reduce (Ansorge 2004) or increase (Maciag et al. 2005) locomotor behaviour in novel environments. Suppressed male sexual behaviour is also common (Iñiguez et al. 2010; Maciag et al. 2005; Rayen et al. 2013; Rodriguez-Porcel et al. 2011). Some studies have pointed to post-natal effects from pre-natal exposure in humans (Hermansen and Melinder 2015; Oberlander et al. 2010).

In the light of the increasing use of fish as model organisms for research on pharmaceuticals in both environmental and clinical contexts, it is important to fill the knowledge gap regarding the effects of developmental exposure to SSRI in fish. In this study, we take the first steps towards acquiring this missing knowledge by quantifying a range of behavioural measures after developmental exposure of three-spine sticklebacks (*Gasterosteus aculeatus*) to a dose of 1.5 µg/l citalopram in the surrounding water. Pilot studies in zebrafish have indicated that the lower limit for concentrations that will produce behavioural alterations in on-going exposure of adult fish is between 0.1 and 1 µg/l. The concentration used in the current study was chosen as a compromise that was likely to obtain useful results without being too far from environmental concentrations. Thus, the chosen concentration of 1.5 µg/l is a concentration which is approximately one order of magnitude higher than is commonly found in polluted waters but which has been found in extreme cases. At the same time it was deemed high enough, compared to the effective range in adult exposures, to be likely to produce an effect. We hypothesised that exposure to the SSRI citalopram during development would cause long-lasting anxiogenic effects similar to what has been observed in rodents. We employ a battery of behavioural tests designed to measure anxiety, aggression, feeding behaviour and locomotor behaviour, further described below. Those tests were choosen because they are well known and characterised tests that measure behaviours known to be affected by serotonin and by exposure to SSRIs.

## Material and methods

### Chemicals and preparation of stock solution

Citalopram (98% purity) was purchased from Sigma-Aldrich and a stock solution was prepared by dissolving 10 mg of citalopram in 1 l of milliQ water. No vehicle was used. The stock solution was stored under dark conditions in a refrigerator. Since the water volume of the exposure aquaria was 6 l, an initial dose of 0.9 ml stock solution was administered to each aquarium, yielding a concentration of 1.5 µg/l. When half the water was subsequently changed, 0.45 ml of stock solution was added to compensate for the amount lost.

### Experimental design and fish maintenance

Sticklebacks were caught at the Askö laboratory in the Trosa archipelago on the Swedish east coast (58° 49.5′N, 17° 39′E) and were transported to the stickleback facility at Stockholm university within 24 h where the fish were kept in 0.5% artificial brackish water and a water temperature of approximately 20 °C. Details of the timing of different events during the study are summarised in Table 1. After acclimatisation seven pairs were mated to produce offspring. The pairs were formed randomly and fish were not chosen by any particular criteria except sexual maturation. The fertilised eggs were taken from the male nest within 24 h of fertilization and allowed to develop in absence of the father to eliminate confounding effects of paternal care. Each of the seven sibling groups was divided into one exposed and one control subgroup, yielding a paired design. All sibling groups hatched during a period of 7 days. Stickleback embryos in the treatment group were exposed to 1.5 µg/l of citalopram dissolved in the ambient water for 30 days while the control group received a corresponding amount of milliQ water, starting at 2 days post-fertilization (dpf). After 1–2 weeks excess fry were pruned to produce groups of 12–18 individuals. When the exposure period was over, the aquaria were emptied and cleaned in order to remove all citalopram. For approximately the first 6 weeks, each subgroup was kept in 6 litre plastic tanks but as they grew bigger, each subgroup was kept in a 50 l aquarium (60 × 30 × 28 cm). The water regime was semi-static; half of the water was changed every second day at which point the aquarium was cleaned from faeces and food residues. After every water change, new citalopram solution or milliQ water was added to compensate for the amount lost. Due to human error, no water samples were taken but the exact same exposure regime that was used in the current paper has consistently yielded measured concentrations of 50–75% of the nominal concentration in previous studies (Olsén et al. 2014; Kellner et al. 2015, 2016). Those studies were performed with adult fish but this discrepancy should not substantially change the concentrations. However, because the exact concentration is not known, the nominal concentrations should be seen as a maximum. The fish were kept on a 16:8 day:night cycle and fed daily to satiation. When small, the fish were fed live *Artemia* nauplii. Since *Artemia* can survive for an extended time in brackish water, it was possible to give the fry a constant access to nauplii. From approximately 50 dpf they were fed daily to satiation with frozen bloodworms or *Cyklops*. At 77–84 dpf the sticklebacks were labelled using VIE (Visible Implant Elastomer, Northwest Marine Technology) tags. Labelling was performed under MS222 anaesthesia. Two strands of elastomer were used, one on each side of the dorsal spines. The elastomer tags denoted sibling group and treatment. One fish died for unknown reasons shortly after tagging. After tagging, both control and exposed fish were pooled in one big aquarium (117 × 107 × 63 cm) where they were allowed to develop in clean water for 7–8 weeks before they were transported to the stickleback facility at Södertörn university for further development and behavioural testing. After the move to Södertörn university the fish were kept in holding tanks with a flow-through system. The water was fresh, aerated tap water and was kept at a temperature of 12–15 °C and the day:night cycle was changed to 8:16. All handling of experimental animals was permitted by the Ethical Committee on Animal experiments in northern Stockholm (dnr N 22/15) and complies with current Swedish law. All behavioural testing took place between 8 a.m. and 1 p.m. and feeding took place between 3 p.m. and 5 p.m. During testing, all staff left the room and the behaviour was recorded on video. The videos were analysed manually and blindly, as the colour tags could only be seen using a specialised lamp. The order of the behavioural tests was scototaxis—novel tank—feeding—aggression. For the novel tank scototaxis and feeding tests, fish were selected randomly from the main holding tank. The fish that had been used for one test was kept separated from the main bulk of fish in order not to test the same fish twice. Once a behavioural test was over, the fish that had been used were transferred back to the main holding tank. Two weeks were allowed to pass between the scototaxis and novel tank test and between the novel tank and feeding tests. For the aggression test, the fish that had been used for the feeding test were simply re-used as they were already in the test aquaria.

**Table 1:** Progression of the study for the whole cohort. Each event is denoted by the number of days after fertilisation of the eggs. All fry hatched within 7 days

Time (days)	Event
0	Fertilisation
1	Removal from nest
2	Exposure start
7–14	Pruning of excess fry
32	Exposure end
41–43	Move to bigger aquarium
Approx. 50	Start feeding bloodworms and *Cyklops*
80 ± 3	VIE tagging, pooling in bigger aquarium
129 ± 3	Transport to Södertörn university
150 ± 3	Scototaxis test start
175 ± 3	Scototaxis test end
190 ± 3	Novel tank test start
200 ± 3	Novel tank test end
214 ± 3	Feeding test start
224 ± 3	Feeding test end
225 ± 3	Aggression test start
226 ± 3	Aggression test end
250 ± 3	Development of nuptial colouring start
330 ± 3	Experiment termination

### Feeding test

Fish were kept solitarily in smaller aquaria (28.5 × 19.8 × 19.6 cm) for one week prior to the feeding test. During this time they continued to be fed daily as before and the aquaria were cleaned every second day. The fish were fed a piece of frozen bloodworms in their home tank. Behaviour was recorded on video for 10 min and was quantified manually at a later point. The number of attacks on food was used as a measure of food intake. In the cases where a fish would spit the food out only to immediately eat it again, only one attack was counted since the secondary ingestion was deemed as food handling. This method has been employed for three-spine sticklebacks previously and is described in more detail in Kellner et al. (2016).

### Aggression

The same fish that were used for the feeding test were used in the aggression test but at least 24 h were allowed to pass between the two tests. On the day of testing, a mirror was carefully lowered into the test aquarium and the behaviour was then recorded on video for 10 min. Five minutes was analysed for each fish by counting the number of bites against the mirror image, starting when the fish first discovered its mirror image. Those fish that did not discover the mirror image for the first five minutes (i.e., in time to yield five minutes of behaviour that could be analysed) were omitted from analysis. Using a mirror image to measure aggression is a common method for quantification of aggressive behaviour and has been used previously for zebrafish (*Danio rerio*) (Ariyomo and Watt 2013), siamese fighting fish (*Betta splendens*) (Kohlert et al. 2012), (*Brachyrhaphis episcopi*) (Archard and Braithwaite 2011), rainbow trout (*Onchorynchus mykiss*) (Holmberg et al. 2011) and the three-spine stickleback (Sebire et al. 2015).

### Novel tank test and locomotor activity

Pilot studies revealed that an appropriate level of stress for the novel tank (NT) test setup was achieved if the fish were carefully netted directly from their holding tank and then transferred to the NT test aquarium, rather than going through a pre-treatment beaker as described by Egan et al. (2009). Two test aquaria (49 × 24 × 25) were run in parallel and the whole procedure was recorded on video. The test aquaria were back-lit by a light rack which was dimmed by a sheet of white paper and had a horizontal line drawn on the outside of the glass, approximately half-way between the bottom and the surface, to divide them into an upper and a lower part. Each fish was recorded on video for 10 min, starting when the fish was dropped into the water. The latency to the first detour to the upper half of the test aquarium as well as the number of and duration of such detours was registered. Locomotor activity was quantified during the last two minutes of the test by counting the number of times that the fish crossed any line in a mesh, superimposed on the video screen. The last two minutes were chosen to as far as possible exclude influence of acute stress on locomotor behaviour. During analysis of the resulting videos it became apparent that despite the efforts to maintain a low stress level, some of the fish were too stressed during testing. Those fish typically displayed a behaviour where they rapidly swam up and down between the surface and the bottom of the tank. Since the logic behind the novel tank test is that time spent in the upper half of the aquarium corresponds negatively to stress and anxiety and those fish did not follow that logic, it was deemed that this behaviour was to be seen as a confounding factor and not relevant for the test. Thus, fish that made 25 or more detours to the surface during 10 min, and where each detour lasted on average less than 10 s, were omitted from analysis of NT behaviour and locomotor activity. The novel tank test is common and has been used for several fish species including zebrafish (Sackerman et al. 2010; Egan et al. 2009) and the three-spine stickleback (Kellner et al. 2016). The method used for quantification of locomotor behaviour in the current study has previously been employed in Kellner et al. (2016).

### Scototaxis

Eighteen liters aquaria were used (39 × 22 × 22 cm), filled to a depth of approximately 16 cm. The aquaria were divided into a black and a white half and had a transparent, removable compartment in the middle. Fish were taken from their home tank and allowed to acclimatise for 5 min in the central, transparent compartment. The transparent section was then removed, allowing the fish to swim freely. Behaviour was recorded for 10 min. During analysis, notes were taken of the number of crossings from the dark to the white side of the aquarium and frequency and duration of such detours. Scototaxis has previously been used for several fish species including zebrafish (Maximino et al. 2010; Blaser and Rosemberg 2012) and pumpkinseed sunfish (*Lepomis gibbosus*) (Brandão et al. 2013).

### Body weight

After completion of the behavioural tests, sexual maturity was induced by changing the day:night cycle to 20:4 and the temperature in the tanks to 20 °C. At approximately 330 dpf and once nuptial colouring had developed in the males, the fish were sacrificed by means of a lethal dose of MS222. Fish were blotted off gently with a paper tissue and then weighed. Sex was determined by gonad inspection.

### Statistics

All results were analysed as generalised linear mixed models using the lme4 package (version 1.0–6) in R (version 3.2.2). For the NT test (including locomotor activity), feeding and scototaxis data, sibling group and date of recording were used as random factors. For the aggression data only sibling group was used as random factor. All analyses dealing with count data (number of crossings, bites against mirror and attacks on food) were analysed assuming a Poisson distribution. All models were checked with q–q plots and residual plots and were found not to be over-dispersed. For time data and weight, a Gaussian distribution was assumed. Testing for effects of sibling groups was done similarly but with sibling group used as fixed factor and treatment as random factor. To extract the means and confidence intervals used in the plots in this study from the model (i.e., for plotting), the effects package (version 2.3-0) in R was used. This package extracts the main effects as well as lower and upper 95% confidence limits. Thus, for all analyses using transformations, the plotted means and confidence intervals are back-transformed model estimates. The Anova function from the car package (version 2.1-1) in R was used to extract *p*-values from the statistical models. Two-tailed *α* < 0.05 was used as a criterion for statistical significance throughout the study.

## Results

### Feeding and weight

**Fig. 1:**
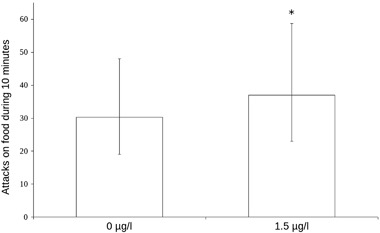
Effect plot from the statistical model of feeding behaviour after developmental exposure to 1.5 µg/l citalopram. *N* = 15 for both groups. Bars denote 95% confidence interval for the fixed factor (treatment) only, asterisk denotes statistical significance (*p* = 0.0012)

Feeding was measured as the number of attacks on food during 10 min and the data from the statistical model are shown in Fig. 1. Thirty-four fish were tested but 4 had to be omitted due to camera malfunction that cut the video short (see Table 2). The mean number of attacks on food was 40.3 (*N* = 15) for the treated fish and 35.7 (*N* = 15) for the control fish. There was a statistically significant effect of treatment on the number of attacks on food (*χ* ^2^ = 10.48, *p* = 0.0012, Fig. 1). Testing the feeding data for effects of sibling group was not deemed relevant because of the few samples in each sibling group.

**Table 2:** Number of samples included in the exposed group and in the control group, and the number of samples omitted from the behavioural tasks in the current study

Task	*N* (control)	*N* (treated)	Omitted
Feeding	15	15	4
Aggression	13	14	7
Novel tank test	48	44	9
Scototaxis	80	62	0

### Aggression

**Fig. 2:**
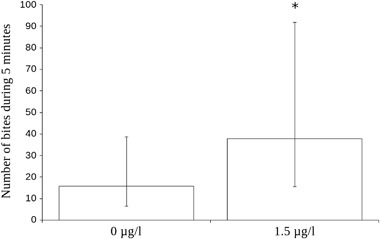
Effect plot from the statistical model of aggressive behaviour in the mirror test after developmental exposure to 1.5 µg/l citalopram. *N* = 13 for the group exposed to 1.5 µg/l citalopram, *N* = 14 for the control group. Bars denote 95% confidence interval for the fixed factor (treatment) only, asterisk denotes statistical significance (*p* < 0.0001)

Effects of developmental citalopram exposure on aggression was tested by lowering a mirror into the fish’ home tank and measuring the number of attacks against the mirror image. The data from the statistical model are shown in Fig. 2. Thirtyfour fish were tested for aggression but seven fish had to be omitted from analysis because they never discovered the mirror or discovered it too late to yield 5 min of video recording for analysis (see Table 2). The mean number of attacks against the mirror for the exposed fish was 38.0 (*N* = 14) while the corresponding number for control fish was 29.4 (*N* = 13), resulting in a statistically significant effect (*χ* ^2^ = 93.36, *p* < 0.0001, Fig. 2). Testing the aggression data for effects of sibling group was not deemed relevant because of the few samples in each sibling group. By coincidence, sibling group 5 was not represented in this dataset.

### Novel tank and locomotor activity

Novel tank behaviour was measured by placing the fish in a non-familiar aquarium and quantifying the latency to the first cross of the horizontal midline, the number of crosses of that line and the total time spent above the line. Three control fish and six exposed fish had to be omitted from analysis because they did not show a response that could reliably be quantified (i.e., they were too stressed and swam erratically back and forth between surface and bottom of the aquarium, see Material and methods). Two fish died as a result of accidental injury during the NT testing phase.

**Fig. 3:**
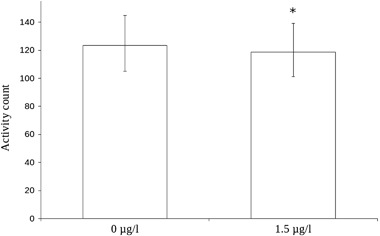
Effect plot from the statistical model of locomotor activity during 2 min in the end of the novel tank test after developmental exposure to 1.5 µg/l citalopram. *N* = 44 for the group exposed to 1.5 µg/l citalopram, *N* = 48 for the control group. Bars denote 95% confidence interval for the fixed factor (treatment) only. Asterisk denotes statistical significance (*p* = 0.043)

The number of fish included in the analysis was 44 for the exposed contingent and 48 for the control (see Table 2). In the NT test, the mean latency to the first cross to the upper half was 237 s for the control fish and 224 s for the exposed fish. There was no significant effect of treatment (*χ* ^2^ = 1.40, *p* = 0.23, data not shown) on the latency to the first cross to the upper half. The mean number of crosses to the upper half of the aquarium was 10.8 for the control fish and 10.3 for the exposed fish. There was no statistically significant effect of treatment on this variable (*χ* ^2^ < 0.01, *p* = 0.98, data not shown). The mean time spent in the upper half of the aquarium was 133.2 s for the control fish and 133.6 s for the exposed contingent. There was no significant effect of treatment (*χ* ^2^ = 0.09, *p* = 0.76, data not shown) on the total time spent in the upper part of the aquarium. Latency to the first cross (*χ* ^2^ = 20.8, *p* = 0.0019) and the number of crosses of the horizontal midline (*χ* ^2^ = 45.46, *p* < 0.0001) were significantly affected by sibling group but there was no effect of sibling group on the total time spent in the upper half (*χ* ^2^ = 11.1, *p* = 0.08, data not shown). The mean locomotor activity count for the exposed fish was 123.7 while the mean activity count for the control fish was 132.5. Locomotor activity was significantly affected by treatment (*χ* ^2^ = 4.09, *p* = 0.043, Fig. 3). Locomotor activity was also significantly different between sibling groups (*χ* ^2^ = 69.73, *p* < 0.001, data not shown).

### Scototaxis

**Fig. 4:**
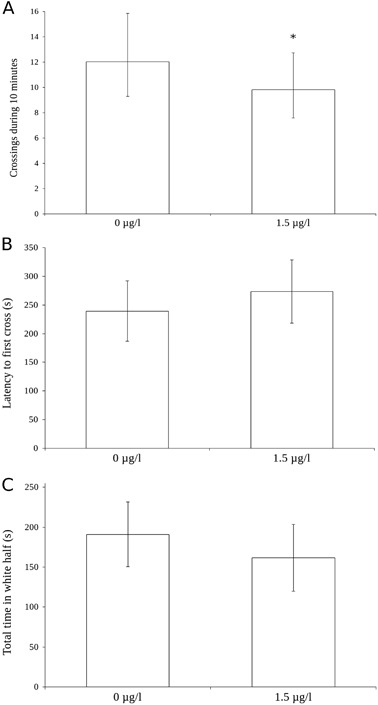
Effect plots from the statistical model of behaviour in the scototaxis (light/dark preference) test after developmental exposure to 1.5 µg/l citalopram. Number of crosses to the whites side (**a**), latency to the first cross to the white side (**b**) and the total time spent on the white side (**c**). *N* = 62 for the group exposed to 1.5 µg/l citalopram, *N* = 80 for the control fish. Bars denote 95% confidence intervals for the fixed factor (treatment) only. Asterisk denotes statistical significance (*p* = 0.0002 for the number of crosses)

**Fig. 5:**
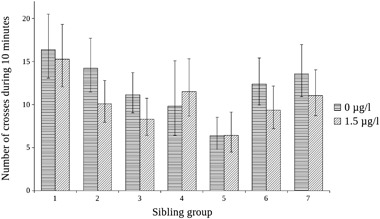
Effect plot from a statistical model of the number of crosses to the white side in the scototaxis test by group and treatment. *N* for the controls are 10 for group 1, 11 for group 2, 18 for group 3, 3 for group 4, 11 for group 5, 12 for group 6, and 14 for group 7. *N* for the exposed fish are 8 for group 1, 11 for group 2, 10 for group 3, 6 for group 4, 6 for group 5, 9 for group 6, and 11 for group 7. Bars denote 95% confidence interval for the fixed factor (sibling group) only

Light/dark preference was tested by placing the fish in an aquarium with a white and a black half and the results are shown in Fig. 4. The mean number of crossings to the white side of the aquarium was 10.7 (*N* = 62) for the exposed fish and 11.8 (*N* = 80) for the control fish (see Table 2). There was a statistically significant effect of treatment on the number of crosses from the white to the black compartment (*χ* ^2^ = 14.00, *p* = 0.0002, Fig. 4). The latency to first cross from the black to the white part of the test aquarium was 270 s (*N* = 62) for the treated fish and 252 s (*N* = 80) seconds for the control fish. The difference was not statistically significant (*χ* ^2^ = 2.10, *p* = 0.15, Fig. 4). The total time spent in the white half was 165 (*N* = 62) for the exposed fish and 189 (*N* = 80) for the control fish. There was no statistically significant effect of treatment on the total time spent on the white side of the aquarium (*χ* ^2^ = 2.49, *p* = 0.11, Fig. 4). There was a statistically significant effect of sibling group on the number of crossings (*χ* ^2^ = 69.84, *p* < 0.0001, Fig. 5), and the total time spent in the white half of the aquarium (*χ* ^2^ = 13.94, *p* = 0.0304, data not shown) but not on the latency to enter the white side (*χ* ^2^ = 8.20, *p* = 0.22, data not shown).

### Weight

Mean final weight in the exposed group was 1.37 g (*N* = 60) and in the control group 1.39 g (*N* = 63). There was no statistically significant treatment*sex interaction (*χ* ^2^ = 2.25, *p* = 0.13, data not shown) or any significant effect of treatment (*χ* ^2^ = 0.33, *p* = 0.56, data not shown) or sex (*χ* ^2^ = 3.3, *p* = 0.07, data not shown) on weight. Weight was significantly affected by sibling group (*χ* ^2^ = 54.6, *p* < 0.001, data not shown).

## Discussion

The current study represents an initial analysis of the effects of developmental exposure to SSRI in fish. Exposure to 1.5 µg/l citalopram during development was found to cause effects on feeding behaviour, aggressive behaviour and locomotor activity in the three-spine stickleback. A decrease in the number of detours to the white side was seen in the scototaxis test. Those effects persisted long after exposure had ceased. To the best knowledge of the authors, this is the first study to show developmental effects of SSRI on adult behaviour in fish. In rodents however, there are several publications indicating potentially adverse effects of developmental SSRI exposure (Ansorge 2004; Iniguez et al. 2014; Iñiguez et al. 2010; Maciag et al. 2005).

The sticklebacks displayed an enhanced feeding behaviour as a result of developmental citalopram treatment. This is contrary to what has been shown for rodents after developmental SSRI exposure as assessed by the latency to feed in a novel environment (Ansorge 2004; Olivier et al. 2011). However, the feeding behaviour in this study was assessed in the home tank of the fish and the variable measured was the number of attacks on food rather than the latency to feed which makes the comparison uncertain. Measuring feeding behaviour in the home tank eliminates the stress of the novel environment. The observations made in this study were also the opposite to what has previously been observed in three-spined sticklebacks exposed to citalopram as adults (Kellner et al. 2015) or in goldfish exposed to the SSRI fluoxetine (Mennigen et al. 2009). In the current study, there was no statistically significant difference in size by the end of the experiment. This is likely due to the high abundance of food during development which may have kept the effects of a potentially elevated food intake from making any significant difference.

Aggression levels were higher in exposed fish than in controls in this study. Effects of developmental exposure to SSRI on aggressive behaviour has not previously been studied in fish but in rodents, several studies show an increase in aggressive behaviour as a result of developmental SSRI exposure (Kiryanova et al. 2016; Svirsky et al. 2016). The results of the current study are in starch contrast to most studies of aggressive behaviour in fish receiving SSRI as adults, as such exposure usually results in reduced aggression (Dzieweczynski and Hebert 2012; Kohlert et al. 2012; McCallum et al. 2017).

Locomotor activity was reduced in the exposed fish compared to controls in the current study. Reduced locomotor activity as a result of developmental SSRI treatment has previously been reported for rodents by Ansorge (2004) while Maciag et al. (2005) reports an increased locomotor activity. In studies of the effects of on-going SSRI exposure on locomotor behaviour in fish, both reduced and enhanced locomotor behaviour has been reported. Thus, an enhanced locomotor activity was observed in adult three-spined sticklebacks during on-going citalopram exposure (Kellner et al. 2016) while fluoxetine exposure suppressed locomotor activity in sheepshead minnow (*Cyprinodon variegatus*) (Winder et al. 2012) and siamese fighting fish (*Betta splendens*) (Kohlert et al. 2012).

There was no significant difference between the exposed fish and the control fish in the novel tank test and only in one variable out of three in the scototaxis test. The effects of developmental citalopram exposure on anxiety-related behaviours were unexpectedly small in light of the pronounced anxiolytic effects seen during exposure in adult three-spine sticklebacks (Kellner et al. 2016) and other species (Sackerman et al. 2010; Maximino et al. 2010) and should be interpreted with caution. The significantly lower number of transitions that the exposed fish performed in the scototaxis test, suggests that they may have been less bold than controls. This is in line with observations in rodent models, where reduced exploratory behaviour in a novel environment (Ansorge 2004) and decreased exploration of a novel object (Rodriguez-Porcel et al. 2011) as a result of developmental SSRI exposure has previously been reported. Kiryanova et al. (2016) however, reports that perinatal exposure to fluoxetine decreased anxiety-like behaviour in mice.

The mechanism behind the observed effects of developmental SSRI exposure is not clear but a commonly reported effect in developmentally exposed adult rats (Maciag et al. 2005; Simpson et al. 2011) is 5-HTT downregulation. The behavioural performance of rats treated during development is indeed similar to that of 5-HTT deficient rats (Ansorge 2004). However, a study on rhesus monkeys indicated that juvenile exposure to the SSRI fluoxetine persistently upregulated 5-HTT expression (Shrestha et al. 2014). Some studies show an altered neuronal morphology in cortical areas due to SSRI exposure during development (Maciag et al. 2005; Simpson et al. 2011; Smit-Rigter et al. 2012), an effect that appears to be mediated by the 5-HT_3_ receptor (Smit-Rigter et al. 2012).

Effects of SSRI on both boldness, locomotor activity, aggression and feeding behaviour are believed to have ecological effects in aquatic ecosystems. Monoamines like serotonin regulate behaviour in response to stressors in different ways depending on the type of stressor faced by the fish. Individuals with high extracellular serotonin levels tend to be more risk taking in the interaction with predators but less aggressive towards conspecifics (Bell et al. 2007). Adult fish under SSRI exposure typically show a behavioural phenotype which is, compared to control fish, less anxious (Kellner et al. 2016; Sackerman et al. 2010), less interested in feeding (Kellner et al. 2015; Mennigen et al. 2010) and less aggressive (Dzieweczynski and Hebert 2012; Kohlert et al. 2012). It is interesting to note that the behavioural phenotype of the sticklebacks in this study is largely opposite to this. They attack food more frequently, are more aggressive and exhibit a lower locomotor activity than control fish. The ecological consequences of such behavioural modulation are difficult to predict and may depend on factors such as predation risk and food abundance. The serotonergic system is evolutionarily ancient and highly conserved throughout vertebrate evolution. The current study therefore further highlights the risks of negative consequences of neonatal and perinatal SSRI exposure not only in fish but also in mammals which have previously been reported (Oberlander et al. 2010).

The current study demonstrates a large variation between sibling groups with regard to feeding, aggression, scototaxis, and the number of detours that the fish did to the upper part of the aquarium as well as the latency to the first detour to the upper half in the NT test. As an example, Fig. 5 shows the variation between sibling groups for the number of crosses to the white side in the scototaxis test. While the genetics underlying behaviour is not in focus for this study, the behavioural variation between sibling groups introduces a higher demand for robustness in the results. Laboratory studies have their limitations, but the use of second generation wild fish in this study is likely to make the study more similar to real field situations and therefore makes the study more environmentally relevant than if a more genetically homogeneous strain had been used. Some within-group variation may have been introduced by the way that the fish were selected for each experiment. It can not be ruled out that since not all fish took part in each experiment, habituation or handling stress may have changed the behaviour of those fish that had been used in a previous experiment. However, a substantial amount of time was allowed to pass between each behavioural test which makes this possibility unlikely.

In conclusion, the current study indicates behavioural effects of developmental exposure to SSRIs in fish which are largely consistent with the results previously reported from developmental exposure in rodents. Those results include increased aggression, reduced locomotor behaviour and possibly an aversive response to novelty. Feeding behaviour was enhanced in this study which is not in accord with previous studies on rodents. So far, research on behavioural and ecological effects of developmental SSRI exposure in fish has been absent. If compared to studies of on-going SSRI exposure however, the current study indicates that in fish, the behavioural consequences of developmental exposure are largely opposite to what has been observed during on-going exposure which complicates the predictions of ecological impact of SSRI pollution.
